# New insights into Nod factor biosynthesis: Analyses of chitooligomers and lipo-chitooligomers of *Rhizobium* sp. IRBG74 mutants

**DOI:** 10.1016/j.carres.2016.08.001

**Published:** 2016-11-03

**Authors:** Véréna Poinsot, Matthew B. Crook, Stéphanie Erdn, Fabienne Maillet, Adeline Bascaules, Jean-Michel Ané

**Affiliations:** a Laboratoire des IMRCP, UMR 5623 Université Paul Sabatier, Toulouse, France; b Department of Bacteriology, University of Wisconsin–Madison, Madison, WI 53706, USA; c Laboratoire des Interactions Plantes–Microorganismes, UMR 441/2594 INRA-CNRS, B.P. 52627, F-31326 Castanet-Tolosan Cedex, France; d Department of Agronomy, University of Wisconsin–Madison, Madison, WI 53706, USA

**Keywords:** Acylation, Arabinosylation, Carbamoylation, Chitooligomers, Fucosylation, Lipo-chitooligomers, Ara, arabinose, Cb, carbamoyl, CO, chitooligomer, dCO, mono-deacetylated chitooligomer, Fuc, fucose, Glc*N*Ac, *N*-acetyl-d-glucosamine, LCO, lipo-chitooligomer, NMe, *N*-methyl

## Abstract

Soil-dwelling, nitrogen-fixing rhizobia signal their presence to legume hosts by secreting lipo-chitooligomers (LCOs) that are decorated with a variety of chemical substituents. It has long been assumed, but never empirically shown, that the LCO backbone is synthesized first by NodC, NodB, and NodA, followed by addition of one or more substituents by other Nod proteins. By analyzing a collection of in-frame deletion mutants of key *nod* genes in the bacterium *Rhizobium* sp. IRBG74 by mass spectrometry, we were able to shed light on the possible substitution order of LCO decorations, and we discovered that the prevailing view is probably erroneous. We found that most substituents could be transferred to a short chitin backbone prior to acylation by NodA, which is probably one of the last steps in LCO biosynthesis. The existence of substituted, short chitin oligomers offers new insights into symbiotic plant–microbe signaling.

## Highlights

• Rhizobia produce chemically substituted, short chitooligomers (COs).
• Deacetylation of the non-reducing GlcNAc is necessary for most substitutions.
• Acylation may be one of the last steps in the biosynthesis of rhizobial lipo-chitooligosaccharides (LCOs).

## Graphical abstract

**Figure:**
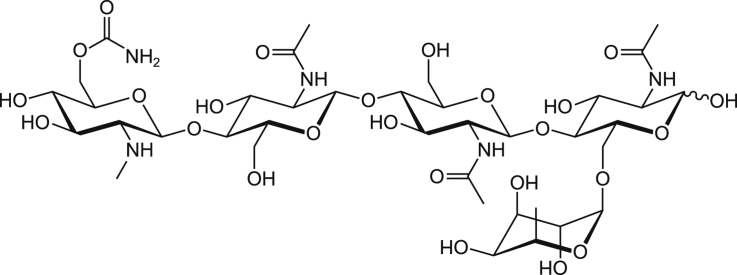

## 1 Introduction

Rhizobia are soil-dwelling, Gram-negative bacteria that can establish a nitrogen-fixing symbiosis with legumes [1]. In this association, legumes develop specialized organs, root nodules, to host the bacteria under low oxygen tensions that are favorable to nitrogen fixation and provide hydrocarbons (malate) to the microorganism to sustain, in particular, the nitrogenase activity. In return, the rhizobia provide ammonium and amino-acids, transformed from atmospheric dinitrogen, to the plant host.

During the early stages of this symbiosis, a molecular dialogue takes place. The plant exudes flavonoids and isoflavonoids into the rhizosphere, and the rhizobia respond by secreting Nod factors [2]. Nod factors are lipo-chitooligomers (LCOs) that are required for nodule development and intracellular infection of the root and are active at very low (nanomolar to picomolar) concentrations. Nod factor structures were extensively studied in the 1990s, and their biosynthesis has also been investigated based on molecular biological tools [3], [4]. The enzymes responsible for Nod factor biosynthesis are encoded by *nod*, *noe*, and *nol* genes, which are located either on symbiotic plasmids or in genomic islands. These genes are found under the control of regulatory elements, called *nod* boxes [5], which are recognized by the transcription factor NodD [6].

While forward and reverse genetics have allowed us to ascertain the role of all the genes involved in Nod factor biosynthesis [7], little is known about the acting order of their corresponding enzymes. To explore this question, we analyzed two classes of molecules, LCOs and chitooligomers (COs), produced by wild-type, overproducing, and mutant strains of *Rhizobium* (*Agrobacterium*) sp. IRBG74 (hereafter IRBG74). This strain was chosen because the annotation of its genome [8] suggested that it would produce a wide array of LCOs, in contrast to more commonly studied strains, such as *Sinorhizobium meliloti* 1021, which produce limited numbers of LCO structures [3]. This strain is also of agronomic interest because it has been shown to colonize rice and increase plant growth and yields [9], [10] in addition to nodulating *Sesbania* species [11], which are recommended for use as a green manure in southeast Asia. With the help of LC-MS/MS experiments, we have been able to deduce the potential step-by-step progression of the biosynthesis of the COs and LCOs of this strain.

## 2 Results and discussion

### 2.1 IRBG74 produces a wide family of LCOs and COs

#### 2.1.1 The LCOs of IRBG74

**Fig. 1:**
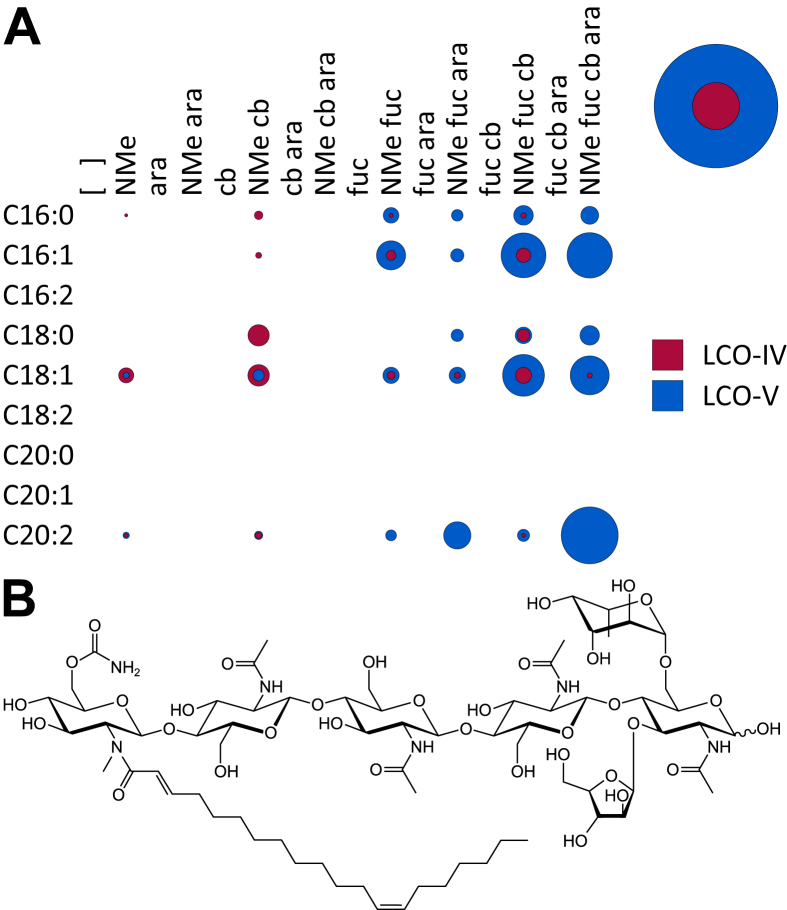
**A**. A bubble matrix of LCOs produced by wild-type *Rhizobium* sp. IRBG74. ara, arabinose; cb, carbamoyl; fuc, fucose; NMe, *N*-methyl. Relative abundances of different chemical species are represented by the size of the bubble where a given row and column overlap. The bubbles at the top right of the figure indicate the overall abundances of the indicated classes of chemicals using the same scale. Fig. 2, Fig. 5, Fig. 6, Fig. 7, Fig. 8, Fig. 9, Fig. 10, Fig. 11 follow this same format. **B**. The structure of the major LCO produced by wild-type *Rhizobium* sp. IRBG74.

Wild-type IRBG74 does not secrete LCOs in substantial amounts. The analysis of 5 L of bacterial exudates was not sufficient for the detection of LCOs by MS (data not shown). Because LCOs are amphipathic, they are most likely to be found in the lipid bilayer [12]. Thus to enhance the production of LCOs, plasmid pA28 [13], which overexpresses the regulatory *nodD*_*1*_ gene of *Sinorhizobium* sp. NGR234, was introduced into IRGB74. The resulting strain secreted amounts of LCOs that were sufficient for analysis. The LCOs secreted into the culture medium by overexpressing strains are not expected to differ significantly from those found in the membranes of wild-type rhizobia [14]. More than 40 different structures were found in the culture supernatant of the *nodD*-overexpressing strain (Fig. 1A). All LCOs exhibited N-methylation of the non-reducing end. Most species were five Glc*N*Ac residues long, carbamoylated, and fucosylated, while arabinosylation was more variable (Fig. 1B). Unsaturated acyl chains were more prevalent, with C16:1 and C18:1 occurring in nearly equal amounts (Table 1). Notably, arabinosylation never occurred in the absence of fucosylation, and more than half of the LCO-IV molecules were carbamoylated without any other decoration.

To check whether the composition of IRBG74's LCOs was affected by regulation using the *nodD* of a phylogenetically distant strain, the native IRBG74 regulator *nodD*_*1*_ and the *nodD*_*2*_–*syrM* regulatory module were each cloned into a plasmid under the control of a constitutive promoter and introduced into wild-type IRBG74. We confirmed by HPLC that the *nodD* genes of *Rhizobium* sp. IRBG74 could be induced by apigenin (Fig. S1) The LCOs produced by overexpression of each of the native *nodD* genes of IRBG74 were very similar to each other but were noticeably different from the profile of the strain overexpressing the *nodD*_*1*_ of *Sinorhizobium* sp. NGR234 (Fig. S2). Because the latter produced a greater diversity of LCO structures, subsequent analyses were conducted exclusively using this overexpression plasmid.

#### 2.1.2 The short COs of IRBG74

**Fig. 2:**
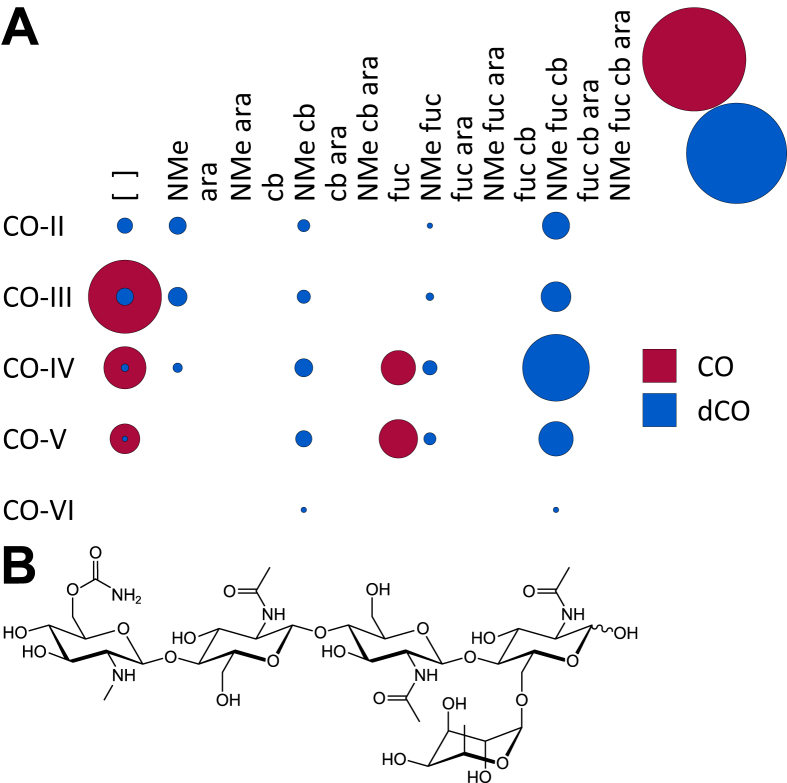
**A**. A bubble matrix of COs and dCOs produced by wild-type *Rhizobium* sp. IRBG74. Abbreviations as in Fig. 1. **B**. The structure of the major dCO produced by wild-type *Rhizobium* sp. IRBG74.

For all the overproducing strains, the bacterial growth medium also contained short COs. Historically these molecules have been discarded during the organic extraction of LCOs, making this the first study to look at them in depth. The COs of IRBG74 ranged in degree of polymerization from two to five Glc*N*Ac residues long, with CO-III and CO-IV being the most common (Fig. 2A). Roughly half of the COs detected in the wild-type exudates were mono-deacetylated at the non-reducing end (Table 1). These were almost always *N*-methylated, carbamoylated, and fucosylated but never arabinosylated (Fig. 2B).

### 2.2 Analysis of *nod* mutants

#### 2.2.1 Overview

**Fig. 3:**
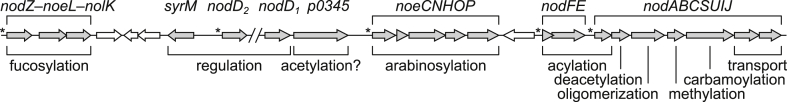
The *nod* genes (depicted in gray) of *Rhizobium* sp. IRBG74 are organized into two clusters, both located on the symbiosis plasmid, pIRBG74a. Above the diagram gene or operon names are indicated. Below the diagram functions are indicated. Asterisks indicate the location of *nod* boxes.

To investigate the order in which the various *nod* genes contribute to the final LCO structures, we developed a collection of mutants in IRBG74. The recent publication of the IRBG74 genome [8] enabled us to identify two clusters of *nod* genes on the symbiosis plasmid, pIRBG74a, and assign putative functions to many of the annotated *nod* genes by homology with previously described *nod* genes (Fig. 3). To ensure that there were no polar effects on expression of downstream genes, we constructed an in-frame deletion of each target gene. In cases where multiple *nod* genes are involved in the biosynthesis of a chemical precursor, we only deleted the transferase responsible for adding the precursor in order to avoid redundancy (*e*.*g*. we only deleted the arabinosyltransferase *noeP* instead of each gene of the *noeCNHOP* arabinosylation operon individually). We started our analysis by deleting the “common” *nod* genes: *nodA*, *nodB*, and *nodC*. These three genes are found in all rhizobia examined so far, with the exception of the photosynthetic bradyrhizobia, which nodulate their *Aeschynomene* hosts without producing Nod factors [15]. These three genes are usually, but not always, found in a single operon, *nodABC*, which has engendered the prevailing model that the products of these genes act first, followed by elaboration with various chemical adducts from the various host-specific *nod* genes.

#### 2.2.2 Chitin synthesis

**Fig. 4:**
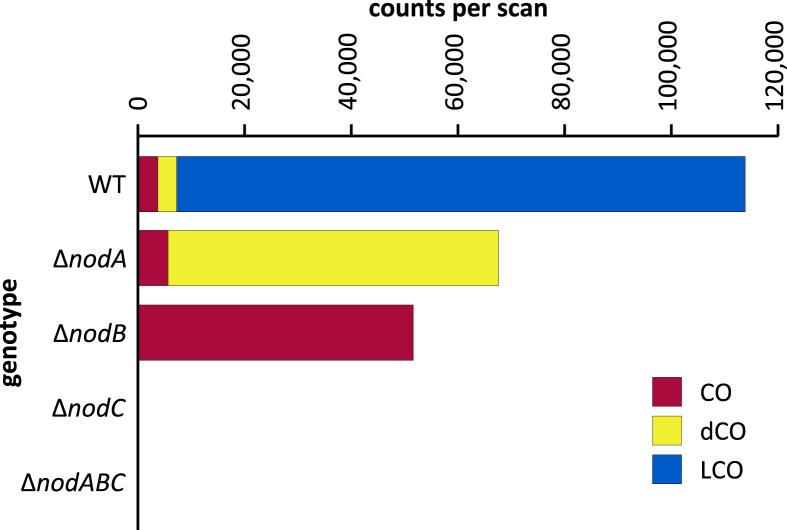
Relative production of COs, dCOs, and LCOs by WT, *nodA*, *nodB*, *nodC*, and *nodABC* strains of *Rhizobium* sp. IRBG74. Values are given in counts per scan (intensity of the detection) assuming that the concentration of each extract was the same (0.1 g/L).

NodC is an *N*-acetylglucosaminyltransferase (EC 2.4.1.–), located in the inner membrane [16], that catalyzes the formation of β1→4 glycosidic linkages between Glc*N*Ac residues from the non-reducing end of the growing chitin chain [17], [18]. It is also involved in determining the degree of polymerization of COs and LCOs [19]. The *nodC* and *nodABC* mutants of IRBG74 do not produce detectable amounts of COs or LCOs (Fig. 4), indicating that biosynthesis of these molecules has been abolished in these strains. To corroborate this result we also tested a *nodC* mutant of *Sinorhizobium meliloti*; again no COs or LCOs were detectable by MS (data not shown).

#### 2.2.3 Mono-deacetylation

**Fig. 5:**
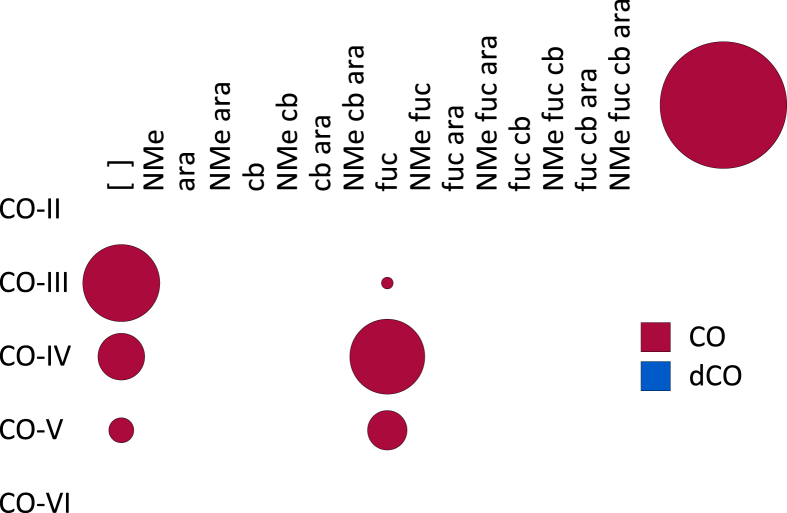
A bubble matrix of COs produced by a *nodB* mutant of *Rhizobium* sp. IRBG74. Abbreviations as in Fig. 1.

NodB is a chitin *N*-deacetylase (EC 3.5.1.–) which removes the acetyl group of only the non-reducing terminal Glc*N*Ac [20]. As expected, the *nodB* mutant of IRBG74 still produces COs, but no longer produces dCOs or LCOs (Fig. 4, Fig. 5). Surprisingly, substantial quantities (∼46%) of CO-IV and CO-V with a fucose on the non-reducing terminal Glc*N*Ac were detected. This composition is similar to the COs produced by wild type (∼50% fucosylated), but less than the LCOs produced by wild type (∼91%) (Table 1). Methylation, carbamoylation, and arabinosylation were never detected. The degree of polymerization of COs was also reduced in the *nodB* mutant, with higher proportions of CO-III and CO-IV than what was observed in the wild type.

#### 2.2.4 *N*-acylation

**Fig. 6:**
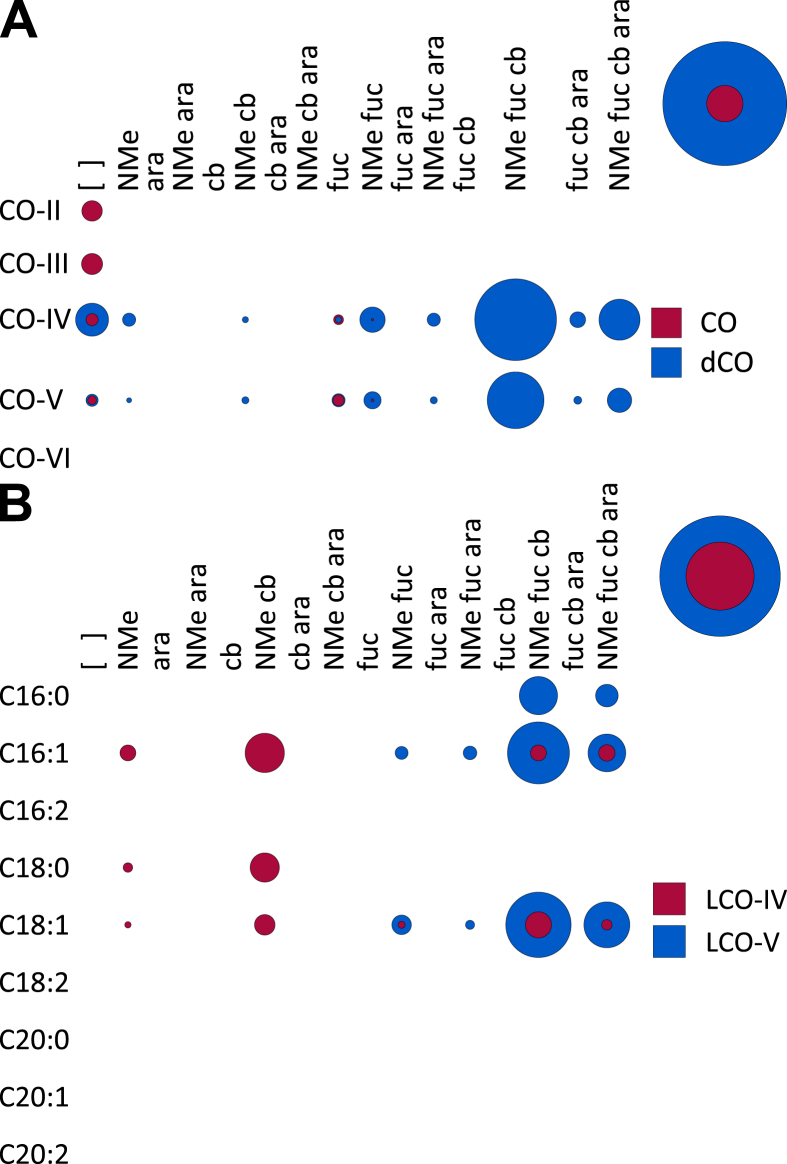
**A**. A bubble matrix of COs and dCOs produced by a *nodA* mutant of *Rhizobium* sp. IRBG74. **B**. A bubble matrix of LCOs produced by a *nodE* mutant of *Rhizobium* sp. IRBG74. Abbreviations as in Fig. 1.

NodA is an *N*-acyltransferase (EC 2.3.1.–) that transfers fatty acids from an acyl carrier protein [21], [22] to the non-reducing terminal Glc*N*Ac of LCOs [23], [24], [25]. The *nodA* mutant of IRBG74 no longer produces LCOs, but it does produce a small distribution of short COs and a wide family of substituted dCOs, principally dCO-IV (Cb,Fuc,NMe) and dCO-V (Cb,Fuc,NMe) (Fig. 4 and Fig. 6A). Consistent with the results from the *nodB* mutant, COs are never *N*-methylated, arabinosylated, or carbamoylated unless they have been deacetylated first, but they can still be fucosylated. The dCOs of the *nodA* mutant are less likely to be *N*-methylated (∼82% vs. 100%) or arabinosylated (∼17% vs. ∼52%) than the LCOs of wild-type (Table 1). However, compared to the dCOs produced by wild-type IRBG74, the rate of substitution increased for every type of substitution in the *nodA* mutant, except arabinosylation (Table 1). To corroborate these results, we also tested a *nodA* mutant of *Sinorhizobium meliloti*. Likewise, this strain no longer produced any LCOs but still produced both COs and dCOs (Fig. S3). Intriguingly, the COs showed a small degree of sulfation (∼5%), which considerably increased in the dCO population (∼63%). This is consistent with previous reports that the sulfotransferase, NodH, utilizes both COs and LCOs as substrates, though LCOs are preferred [26], [27], [28]. It is also worth noting that acetylation of the non-reducing terminal Glc*N*Ac was never observed in the absence of sulfation (Fig. S3), suggesting that the former process may be dependent on the latter.

NodE is a specific acyl carrier protein that, along with NodF, a β-ketoacyl ACP synthase I (EC 2.3.1.41), is involved in the synthesis of polyunsaturated acyl groups which are then transferred to the non-reducing terminal Glc*N*Ac of LCOs [29]. Deletion of *nodE* in IRBG74 predictably results in the complete loss of LCOs with polyunsaturated acyl groups and of LCOs with acyl chains longer than 18 carbons (Fig. 6B). There was also a mild decline in the proportion of arabinosylated LCOs (Table 1).

#### 2.2.5 Fucosylation

**Fig. 7:**
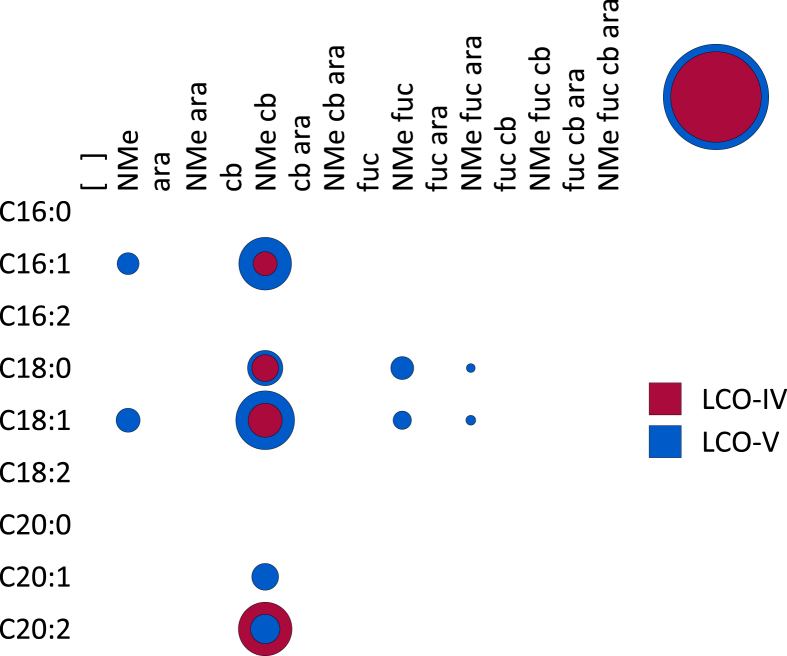
A bubble matrix of LCOs produced by a *nodZ* mutant of *Rhizobium* sp. IRBG74. Abbreviations as in Fig. 1.

Because we observed fucosylated COs produced by the *nodB* mutant of IRBG74, we next turned our attention to *nodZ*. NodZ is an α-1,6-fucosyltransferase (EC 2.4.1.–) that fucosylates C-6 of the reducing terminal Glc*N*Ac of Nod factors [30]. In the *nodZ* mutant fucosylation was not completely abolished—about 5% of LCOs were still fucosylated (Table 1). A similar result was observed for a *nodZ* mutant of *Azorhizobium caulinodans* ORS571 [31]. The persistence of fucosylation in the absence of NodZ may be due to the presence of an alternative, albeit less specific, fucosyltransferase in the IRBG74 genome or may indicate that the activation energy of fucose reacting with C-6 of the terminal Glc*N*Ac of (L)COs is low enough that this reaction naturally occurs at a low level. No LCOs bearing arabinose groups directly on the chitin backbone could be detected unless there was also a fucose present (Fig. 7), which was also observed for a *nodZ* mutant of *Azorhizobium caulinodans* ORS571 [31]. N-methylation was still universal and carbamoylation still common in the *nodZ* mutant.

#### 2.2.6 *N*-Methylation

**Fig. 8:**
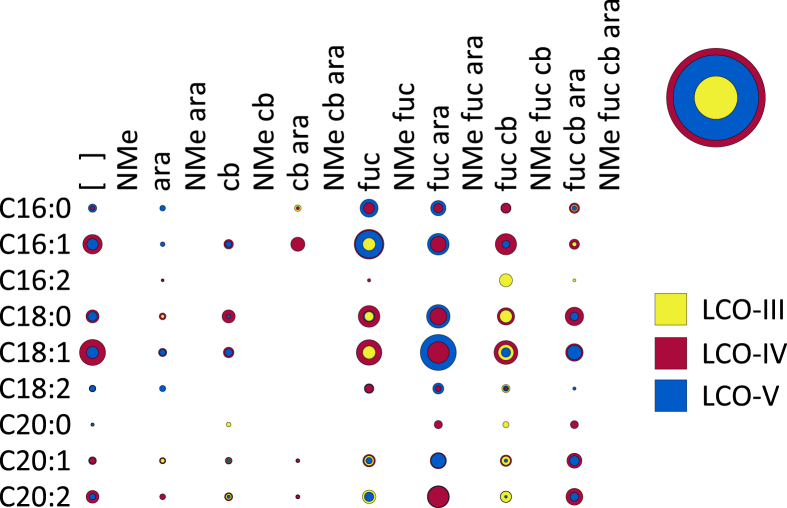
A bubble matrix of LCOs produced by a *nodS* mutant of *Rhizobium* sp. IRBG74. Abbreviations as in Fig. 1.

NodS is a SAM-dependent methyltransferase (EC 2.1.1.–) that *N*-methylates the non-reducing terminal Glc*N*Ac of Nod factors [32], [33]. As expected, the LCOs of the *nodS* mutant were strictly unmethylated at the non-reducing terminal Glc*N*Ac (Fig. 8). Surprisingly, the LCOs of the *nodS* mutant showed a low degree of carbamoylation. In wild-type IRBG74, 80% of the LCOs are carbamoylated whereas in the *nodS* mutant this was reduced to about 30% (Table 1). Arabinosylation and fucosylation were unaffected. There was also a reduction in the degree of polymerization compared to wild type (Table 1), similar to what others have observed [34].

#### 2.2.7 Carbamoylation

**Fig. 9:**
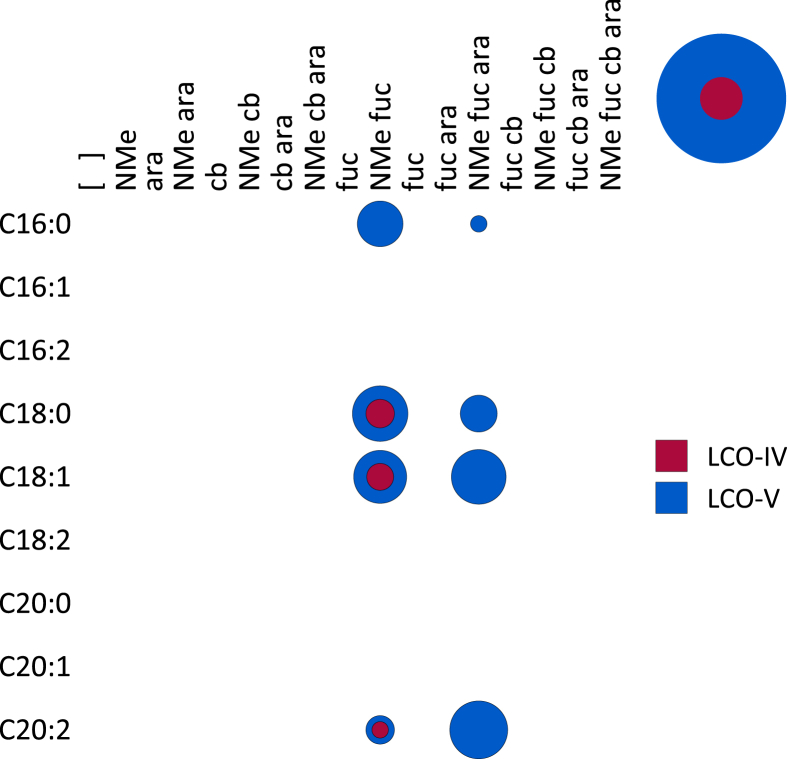
A bubble matrix of LCOs produced by a *nodU* mutant of *Rhizobium* sp. IRBG74. Abbreviations as in Fig. 1.

NodU is an *O*-carbamoyltransferase (EC 2.1.3.–) that carbamoylates C-6 of the non-reducing terminal Glc*N*Ac of Nod factors [34]. A second *O*-carbamoyltransferase found in some rhizobia (but not IRBG74), NolO, carbamoylates C-3 of the non-reducing terminal Glc*N*Ac of Nod factors [35]. The LCO profile of the *nodU* mutant of IRBG74 was similar to wild type except that no carbamoylated species were observed (Table 1). Also, in the absence of carbamoylation, LCO-IV species were never arabinosylated (Fig. 9).

#### 2.2.8 Arabinosylation

**Fig. 10:**
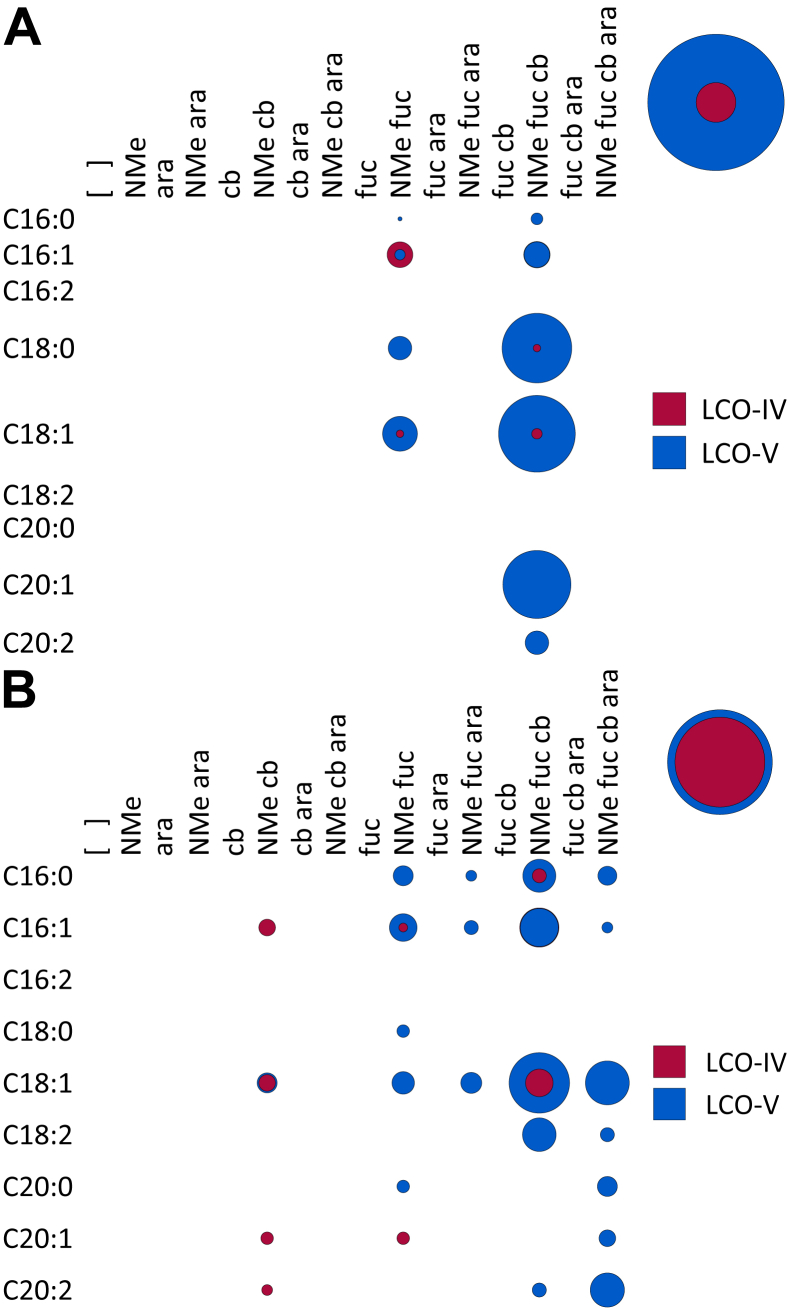
**A**. A bubble matrix of LCOs produced by a *noeP* mutant of *Rhizobium* sp. IRBG74. Abbreviations as in Fig. 1. **B**. A bubble matrix of LCOs produced by a *noeN* mutant of *Rhizobium* sp. IRBG74. Abbreviations as in Fig. 1.

NoeP was first described as part of the *noeCHOP* operon of *Azorhizobium caulinodans* ORS571, which is involved in d-arabinofuranosylation of C-3 of the reducing-end Glc*N*Ac of Nod factors [31]. Later work on orthologs in *Mycobacterium tuberculosis* established that *noeC*, *noeH*, and *noeO* were involved in three of the four steps in the biosynthesis of decaprenylphosphoryl arabinofuranose [36], [37]. No orthologs of *noeP* were detected in *Mycobacterium tuberculosis* [36], suggesting it encodes the 3-*O*-arabinosyltransferase (EC 2.4.2.–). When *noeP* is deleted in IRBG74, no arabinosylated LCOs are produced (Fig. 10A). Fucosylation, carbamoylation, and methylation of LCOs are unaffected (Table 1).

The arabinosylation operon of IRBG74 includes an additional gene, *BN877_p0343*, which here we propose to name *noeN*. The *noeN* gene may encode the decaprenylphosphoryl-β-d-5-phosphoribose phosphatase (EC 3.1.3.–) not identified by Mikusová et al. [37] Deletion of *noeN* in IRBG74 reduces, but does not abolish, arabinosylation of LCOs (Fig. 10B), suggesting that the reaction catalyzed by NoeN (possibly conversion of *trans*,*octacis*-decaprenylphospho-β-d-ribofuranose 5-phosphate to *trans*,*octacis*-decaprenylphospho-β-d-ribofuranose) can occur spontaneously or else can be inefficiently performed by some other enzyme produced by IRBG74. As with the *noeP* mutant, fucosylation, carbamoylation, and methylation of LCOs are unaffected in the *noeN* mutant (Table 1).

#### 2.2.9 BN877_p0345

**Fig. 11:**
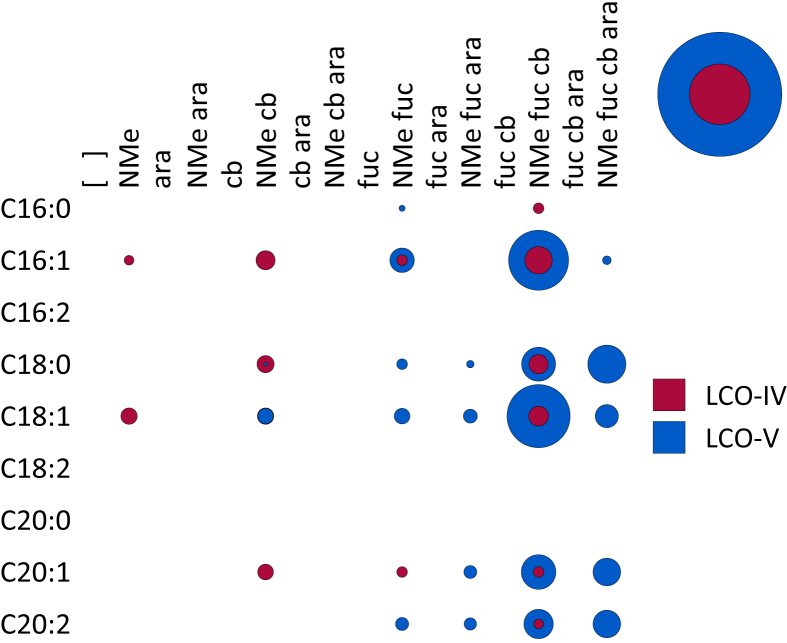
A bubble matrix of LCOs produced by an *IRBLv2_p0345* mutant of *Rhizobium* sp. IRBG74. Abbreviations as in Fig. 1.

Adjacent to the *nodD*_*1*_ gene of IRBG74 is a gene systematically annotated as *BN877_p0345* (hereafter simply *p0345*; Fig. 4) that encodes a putative acyltransferase or acetyltransferase (EC 2.3.–.–). It shares 28.5% identity with *noeT* of *Neorhizobium galegae* HAMBI 1141 and 28.1% identity with *hsnT* of *Rhizobium leguminosarum* sv. *trifolii* ICC105 at the amino acid level. NoeT is responsible for *O*-acetylation of C-3 of the Glc*N*Ac residue immediately adjacent to the non-reducing terminal Glc*N*Ac [38], [39]. The *hsnT* gene is frequently found associated with *nodF* and *nodE*, suggesting that it may encode an acyltransferase [40], [41]. In the *p0345* mutant we did not observe the complete or near complete eradication of any LCO modification (Fig. 11), so its function is still unknown. We did, however, observe a lower degree of arabinosylation in the *p0345* mutant (∼16%) compared to the wild type (∼52%) (Table 1).

## 3 Conclusion

**Scheme 1:**
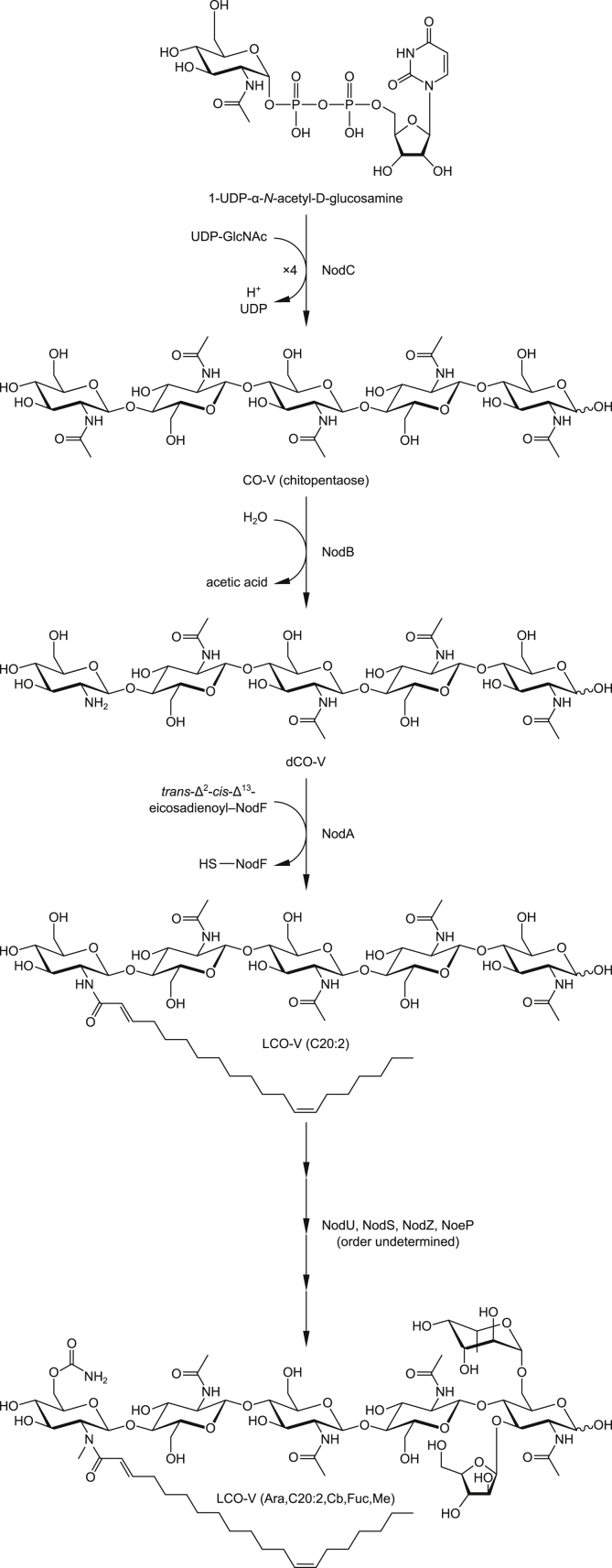
The traditionally assumed model of LCO biosynthesis, with the major LCO of *Rhizobium* sp. IRBG74 as the example. NodC, NodB, and NodA act in sequence to create the LCO backbone, followed by the addition of various decorations in no particular order.

It has generally been assumed (see, for example, Jabbouri et al.^34^), based on the fact that *nodA*, *nodB*, and *nodC* occur in a single operon in most rhizobia examined, that the basic LCO structure is synthesized first (chain elongation → deacetylation → acylation), and then other chemical substituents are added later (Scheme 1). Our results suggest that this view may be erroneous.

**Scheme 2:**
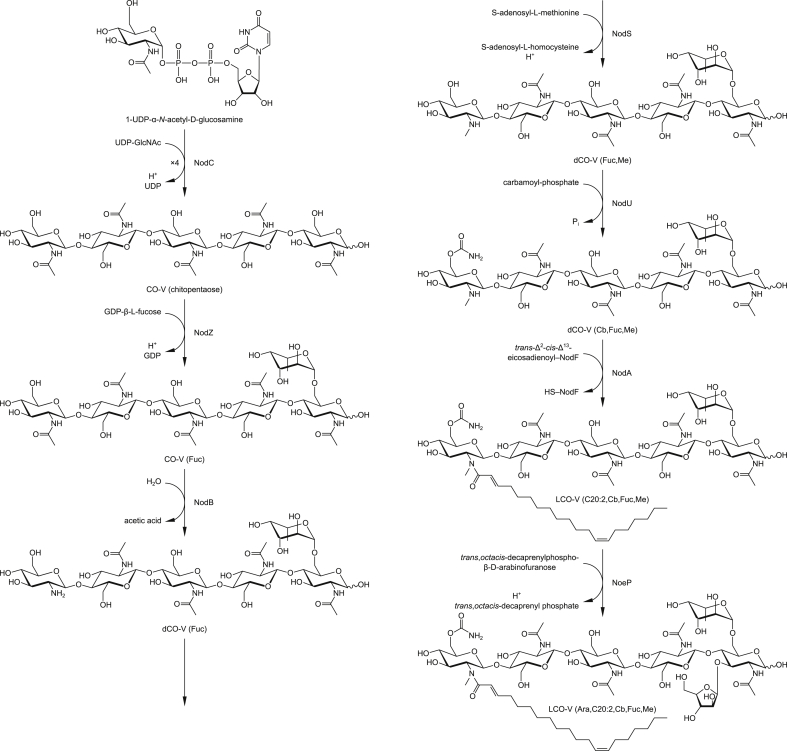
An alternative model of LCO biosynthesis, as suggested by the MS data, with the major LCO of *Rhizobium* sp. IRBG74 as the example.

NodC necessarily acts first to polymerize UDP-Glc*N*Ac into chitin, mainly CO-IV and CO-V (Fig. 4). However, after this point our results suggest that the synthesis diverges from the traditional view (Scheme 2). The next step appears to be fucosylation, as evidenced by the fact that roughly half of the COs (principally as CO-IV) produced by wild-type IRB74 (Fig. 3) and the *nodB* mutant (Fig. 5) are fucosylated. This is consistent with the observations of Quinto et al. that NodZ prefers COs over LCOs as substrates [42]. CO-III (Fuc) and LCO-III (Fuc) were rarely observed, suggesting that NodZ prefers CO-IV as its substrate. Furthermore, since the percentage of LCO-V molecules declines in relation to the percentage of LCO-IV molecules in the *nodZ* mutant (Fig. 7), NodC may normally terminate the chitin chain at four residues, but fucosylation by NodZ could promote one more round of polymerization. However, this is a complicated trait since the degree of polymerization is also diminished in the *nodA*, *nodB*, and *nodS* mutants (Table 1), all of which are impaired in enzymes that act at the non-reducing end of the chitin chain, where polymerization occurs. All other additions to the chitin backbone are strictly dependent on mono-deacetylation (Fig. 5).

*N*-methylation occurs after mono-deacetylation (Fig. 5), as has been suggested previously [18], [33]. No un-*N*-methylated compounds were detected from the wild-type strain (Fig. 2; Fig. 3). Furthermore, COs were never *N*-methylated, either in the wild-type strain (Fig. 3) or the *nodA* (Fig. 6) or *nodB* (Fig. 5) mutants. Carbamoylation by NodU is generally subsequent to N-methylation by NodS as carbamoylation is reduced (but not abolished) in the *nodS* mutant (Fig. 8) and rarely occurs independently of N-methylation in the *nodA* mutant (Fig. 6), whereas N-methylation is unaffected in the *nodU* mutant (Fig. 9). This result would suggest that NodU is adapted to use *N*-methylated dCOs as substrates but is non-specific enough that it can utilize non-*N*-methylated dCOs, albeit at lower efficiency. It should be noted that the reduced activity of NodU in the *nodS* mutant could, instead, be due to a reduction in *nodU* expression (despite our use of an in-frame deletion, which would be expected to circumvent such polar effects). However, this interpretation does not account for the phenotype in the *nodA* mutant.

Arabinosylation and acylation most likely occur at the very end of LCO biosynthesis in IRBG74. Arabinosylation clearly takes place after fucosylation since arabinosylated, non-fucosylated LCOs and COs are never detected in wild-type IRBG74 (Fig. 2, Fig. 3) or any of its mutants (except for a small fraction seen in the *nodS* mutant). This result is in contrast to *Azorhizobium caulinodans* ORS571, where arabinosylation can readily occur independently of fucosylation [43]. Given the data, it is possible that in IRBG74 arabinosylation occurs on the fucose rather than on C-3 of the reducing terminal Glc*N*Ac. To determine this conclusively will require enzyme treatment or linkage analysis in comparison with standard compounds. Arabinosylated COs are never detected in wild-type IRBG74 (Fig. 3) or the *nodB* mutant (Fig. 5), which would suggest that arabinosylation cannot occur unless the CO has been deacetylated. Arabinosylation is independent of carbamoylation (Fig. 9) and N-methylation (Fig. 8) and does not appear to affect those processes in turn (Fig. 10). The fact that carbamoylation, fucosylation, and methylation of dCOs increases in the *nodA* mutant suggests that NodA preferentially acylates decorated COs, positioning it later in the process of biosynthesis. The fatty acids employed by NodA for acylation of LCOs were fairly consistent across the mutant collection (with the exception of the *nodE* mutant), suggesting that the fatty acid species transferred by NodA are independent of all other LCO chemical substituents.

We have reached these conclusions by observing concomitant perturbations in the products of intact proteins when one such protein is removed from the biosynthetic pathway. This approach has been employed to interrogate the biosynthetic sequence of such diverse compounds as glycopeptide antibiotics [44], [45], carbapenem antibiotics [46], carbazole alkaloids [47], indole alkaloids [48], pipolythiodioxopiperazines [49], meroterpenoids [50], xanthones [51], prodiginines [52], fumonisins [53], and rhizobitoxine [54]. In many of these cases, the mutational analyses and spectral analyses were validated using complementary techniques. Similar inferences have been made by other authors about the order of LCO biosynthesis. Based on relative abundance of LCO species, Olsthoorn et al. opined that N-methylation of the non-reducing terminal Glc*N*Ac is inhibited by fucosylation of the Glc*N*Ac immediately adjacent to the non-reducing terminal Glc*N*Ac and that the unidentified α-(1 → 3) fucosyltransferase responsible for this reaction preferentially acts on pentamers [55]. A similar preference for pentamers was also proposed for the *O*-acetyltransferase, NodX [56].

The order of LCO biosynthesis presented in Scheme 2 is the primary pathway suggested by our data. However, there is some evidence of flexibility in particular instances (such as the possibility of NodU carbamoylating dCOs prior to N-methylation, mentioned above). For example, there may be some variability in the working order of NodZ and NodB since the LCOs of wild type (∼91%) and the dCOs of the *nodA* mutant (∼84%) show a greater extent of fucosylation than the *nodB* mutant (∼46%; see Table 1), suggesting that NodZ may have higher specificity for dCOs or that NodB may preferentially use fucosylated COs. Another point of flexibility is in the order of arabinosylation and acylation. Arabinosylation is still detected (though slightly reduced: ∼17% in the *nodA* mutant vs. ∼52% in WT; see Table 1) on the COs of the *nodA* mutant, suggesting that arabinosylation may not be entirely dependent on acylation. Alternatively, arabinosylated COs could be a favored substrate for NodA, resulting in efficient conversion to of these species to LCOs when *nodA* is intact. Ultimately it will be necessary to test the purified enzymes *in vitro* for their substrate specificity to resolve this question.

Two more caveats must be mentioned. First, when enzymes are removed, intermediates can build up and push a reaction in an unexpected direction due to the imbalance of reactants and products. Such a scenario would lead to erroneous conclusions about wild-type LCO biosynthesis. Second, our interpretation of the data rests on the assumption that the activity of the LCO biosynthetic enzymes is, for the most part, irreversible. However, our model (Scheme 2) must necessarily be adjusted if any of these enzymes (or some other unidentified enzyme) efficiently catalyzes the reverse reaction. For example, if NodU catalyzes both the forward and reverse reactions (carbamoylation and de-carbamoylation), then it may be that N-methylation sterically inhibits the reverse reaction, resulting in the accumulation of carbamoylated, *N*-methylated species (Fig. 2, Fig. 3). In this scenario the *nodS* mutant would exhibit lower levels of carbamoylation because NodU is no longer inhibited from catalyzing the reverse reaction (Fig. 8). Furthermore, our model (Scheme 2) is based on the apparently preferred substrates for the LCO biosynthetic enzymes, and so it can only, at best, infer the favored biosynthetic sequence. The fact that most of the enzymes examined were still active to some degree in the various mutant backgrounds tested (the only clear exceptions being the total absence of most substitutions in the *nodB* strain and the abolishment of arabinosylation in the *nodZ* strain) suggests some degree of substrate flexibility (*i*.*e*. LCO biosynthesis is not a strictly stepwise, linear biosynthetic pathway). It must also be acknowledged that the diversity of CO and LCO species produced belies a certain laxity in the biosynthetic sequence. Whether our proposed model (Scheme 2) is truly the primary pathway and/or whether there is extensive cross-feeding between many possible pathways in wild-type IRBG74 (or other rhizobia) remains to be demonstrated.

Ultimately, additional analyses, such as using purified enzymes and purified substrates, heterologous expression, rescue experiments, and/or specific labeling (*e*.*g*. using stable isotopes), will be required to settle these questions and validate the results from the mutational analyses. For example, Quinto et al. [42] and Quesada-Vincens et al. [57] independently used a radiolabeled substrate to demonstrate the preference of NodZ for COs over LCOs. It has also previously been shown, by quantitative substrate analysis, that the preferred substrate of NodL, an *O*-acetyltransferase that acetylates C-6 of the non-reducing terminal Glc*N*Ac, is dCOs [58], [59], [60]. On the other hand, NoeE, a sulfotransferase that sulfurylates C3 of the 2-*O*-methylfucose of fucosylated Nod factors, and NolL, an acetyltransferase that acetylates C4 of the 2-*O*-methylfucose of fucosylated Nod factors, have been shown, using purified substrates, to act after fucosylation [61] and after acylation [62], [63]. It has also been shown, using purified enzymes, that NodS is inactive on NodL products, but not *vice versa* [64]. Heterologous expression has been used to show that NodA can exhibit some degree of specificity in the fatty acids it will transfer to LCOs [25], [65] and that NodH and NodL are capable of, respectively, sulfating and acetylating dCOs [66]. Based on our data, it is not unreasonable to anticipate that a preference for dCOs might be shown for the majority of Nod-factor-modifying enzymes of IRBG74.

The most remarkable observation from this study is that short dCOs can be substituted with many of the same decorations as LCOs in rhizobia. This may be a result of acylation being one of the final steps (rather than one of the early steps) in the biosynthesis of LCOs. We show here that short, decorated dCOs are produced by two different species of rhizobia, *Rhizobium* sp. IRBG74 (Fig. 2; Fig. 6) and *Sinorhizobium meliloti* 1021 (Fig. S3), which suggests that it may be the case for most, if not all, rhizobia. In wild-type rhizobia the majority of LCOs assimilate into the bacterial membrane [12], but hydrophilic COs and dCOs would be capable of diffusing across the membrane and into the extracellular milieu, where they can be readily detected by other organisms. For example, short COs have been shown to induce calcium spiking in soybean [67], *Medicago truncatula* [68], and rice [69]; to induce flavonoid production in *Medicago truncatula* [70]; to induce cortical cell divisions in vetch [71] and transgenic clover [72]; to perturb auxin flow in clover [73]; and to stimulate early nodulin expression in soybean [74]—all requisite steps in the formation of a root nodule. These observations, coupled with the data presented here, suggest that decorated COs may be hitherto unrecognized signaling molecules between plants and microbes, including rhizobia.

## 4 Experimental section

### 4.1 Microbiological techniques

#### 4.1.1 Culture techniques

For CO and LCO production, cells were grown overnight in 60 mL of minimal medium (10 g sodium glutamate, 0.22 g K_2_HPO_4_, 0.1 g MgSO_4_, 0.1 g NaCl, 20 mg FeCl_3_, and 0.5 mg biotin per L of water) at 30 °C at 200 rpm and added to 6 L of fresh medium. After induction with 500 nM apigenin, cells were grown for 16 h at 30 °C at 200 rpm.

For strain and plasmid construction, *E*. *coli* and *Rhizobium* sp. IRBG74 were grown on LB agar at 37 °C and 30 °C, respectively. When necessary, the LB agar was supplemented with one or more of the following: gentamicin (Gm, 50 ng/μL for *E*. *coli* and 150 ng/μL for *Rhizobium*), spectinomycin (Sp, 50 ng/μL), sucrose (10%), tetracycline (Tc, 10 ng/μL).

#### 4.1.2 Strain and plasmid construction

Bacterial strains and plasmids are described in Supplementary data, Table S1 (see also Fig. 1A). All custom primers were ordered from Integrated DNA Technologies and are described in Supplementary data, Table S2. To ensure that downstream genes were unaffected in their expression, in-frame deletions of *nod* genes were created using the method of Quandt and Hynes [75]. Briefly, approximately 500 bp of upstream and downstream sequence were amplified and spliced together by overlap-extension polymerase chain reaction using *Pfu* polymerase and then cloned into the *sacB* suicide vector, pJQ200SK [75], using Gateway^®^ cloning (Invitrogen). The resulting deletion constructs were introduced into IRBG74 by triparental mating, followed by selection on LB-Sp-Gm. Integration of the deletion construct into the correct site was confirmed by PCR, and then de-integration was selected for by plating on LB-sucrose. Colonies that were resistant to sucrose and sensitive to gentamicin were checked by PCR for deletion of the target gene. To construct the plasmids overexpressing the *nodD* genes of IRBG74, the coding region along with the predicted ribosome binding site was amplified using *Pfu* polymerase and then cloned into the expression vector, pRF771 [76], using Gateway^®^ cloning (Invitrogen). The resulting expression constructs were introduced into IRBG74 by triparental mating followed by selection on LB-Sp-Tc.

### 4.2 Purification

#### 4.2.1 Purification of LCOs

LCOs were extracted from filtered culture supernatants by butanol extraction, as described previously [77]. Purification was first performed by HPLC on a semi-preparative C18 reverse phase column (7.5 × 250 mm, Spherisorb ODS2, 5 μm) with 10 min in isocratic solvent A (water/acetonitrile 80:20 v/v), followed by a linear gradient from solvent A to solvent B (100% acetonitrile) for 40 min at a flow rate of 1 mL min^−1^, monitoring the UV absorption at 206 nm and 220 nm.

#### 4.2.2 Purification of COs

For purification of COs, the butanol-extracted water phase was first concentrated on a rotative evaporator at 40 °C under 40 mBar. The concentrated phase was then freeze-dried. The powder was then resuspended at 10^−4^ M in water, sonicated, centrifuged, and placed in an O dialyzing tube for an overnight desalting with a cutoff of 1000 Da. (COs are not easily soluble in water at the concentrations used here, so they form aggregates and are not lost.) Finally, the desalted solution was set to 20% acetonitrile (ACN) and transferred to a glass vial for LC/MS analyses.

### 4.3 Mass spectrometry

#### 4.3.1 MS analysis on Q-Tof

HPLC-produced fractions were analyzed using an ESI-QqToF Ultima apparatus (Waters, Milford, Massachusetts, USA) using direct infusion. Spectra were recorded in both the positive and the negative mode. Peaks detected in the expected range (*m*/*z* 1000–1500 for the single-charged species or 600–700 for the double-charged species) were submitted to MS/MS analysis, and exact masses were recorded to confirm that they were LCOs. Energies were as follows: probe, 3 kV; cone, 100 V; Rf, 70 V; collision cell, 15 V for MS and 30 V for MS/MS. Collision gas: argon. Direct inlet: solvent AcCN/H_2_O 1:1, 1% acetic acid, rate: 10 μL min^−1^. Concentrations were about 10^−4^ mol L^−1^.

#### 4.3.2 LC/MS analyses on Q-trap

##### 4.3.2.1 LCO analyses

For analyses of LCOs, butanol extracts or pre-purified HPLC fractions were submitted to UPLC-(MS/)MS analyses. The column was an Acquity C18 BEH 150 × 2.1 mm, 1.7 μm. The gradient was 20% ACN in water with 0.1% formic acid (2 min), up to 100% ACN in 9 min, back to 20% in 1 min. The UPLC was a Dionex Ultimate 3000 system. Injected volume was 10 μl, concentrations were around 10^−5^ M. The mass spectrometer was an AB Sciex Qtrap 4500 device. Analyses were performed in the positive electrospray mode with curtain gas. The compounds were well separated and presented no matrix effect as determined by standard spiking assays. The area of the protonated monoisotopic molecular ion of each species was measured and reported. Quantification was approximate due to the limited availability of standard compounds necessary for a more precise analysis. We assume that the responses of structurally related compounds are similar.

###### 4.3.2.1.1 EMS experiments

For enhanced mass spectrometry (EMS) experiments, the linear trap quadrupole (LTQ) was used to accumulate signal over 300 ms in order to enhance the intensity of the molecular ions detection. Then the scan occurred from 700 to 1900 *m*/*z* at a scan rate of 10,000 scans s^−1^. Data were registered in continuum mode. Protonated molecular ions were analyzed for retentions times ranging from 5 to 8 min.

###### 4.3.2.1.2 EPI experiments

For enhanced product ions (EPI) experiments, which provide a better detection of daughter ions, analyses were performed in enhanced product ion mode. Q1 was set to the parent ion mass and Q3 was in LTQ mode, as in the EMS experiment. Collision energy ramped from 10 to 35 V.

###### 4.3.2.1.3 MRM experiments

For multiple reaction monitoring (MRM) experiments, which were used for the detection of minor compounds that cannot be seen directly on the ESI/MS spectra, up to 30 transitions were programmed on the system with two transitions for a single protonated molecular ion. The collision energy was optimized and ranged from 20 to 30 V, depending on the sugar backbone length. The compounds were assumed to be present when the two transitions were present at the same retention time.

##### 4.3.2.2 CO analyses

For analyses of COs, the dialyzed fractions were subjected to HPLC-MS/MS analyses. The column was a Hypercarb 150 × 2.1 mm, 3 μm. The gradient was 0% ACN in water with 0.1% acetic acid (1 min), up to 100% ACN in 40 min, back to 0% in 2 min. The UPLC was a Dionex Ultimate 3000 system. The injected volume was 10 μl, concentrations were around 10^−4^ M. The mass spectrometer was an AB Sciex Qtrap 4500 device. Analyses were performed in the positive electrospray mode with curtain gas. The compounds were well separated and presented no matrix effect as determined by standard spiking assays. The area of the protonated monoisotopic molecular ion of each species was measured and reported. Quantification was approximate due to the limited availability of standard compounds necessary for a more precise analysis. We assume that the responses of structurally related compounds are similar.

###### 4.3.2.2.1 MRM experiments

For MRM experiments, up to 30 transitions were programmed on the system with two transitions for a single protonated molecular ion. The collision energy was optimized and ranged from 20 to 30 V, depending on the sugar backbone length. The compounds were assumed to be present when the two transitions were present at the same retention time.

## Conflict of interest

None declared.

## Figures and Tables

**Table 1:** Relative percentages of various features of COs and LCOs produced by *Rhizobium* sp. IRBG74 and various *nod* mutants.

	WT LCOs	WT COs	*nodC* COs	*nodZ* LCOs	*nodB* COs	*nodS* LCOs	*nodU* LCOs	*nodA* COs	*nodE* LCOs	*noeP* LCOs	*noeN* LCOs	*p0345* LCOs
II	0.0%	7.0%	0.0%	0.0%	0.0%	0.0%	0.0%	2.5%	0.0%	0.0%	0.0%	0.0%
III	0.0%	34.3%	0.0%	0.0%	37.8%	9.9%	0.0%	2.7%	0.0%	0.0%	0.0%	0.0%
IV	12.6%	39.1%	0.0%	42.4%	48.6%	51.6%	9.8%	66.0%	24.2%	7.8%	19.1%	19.3%
V	87.4%	19.3%	0.0%	57.6%	13.6%	38.6%	90.2%	28.9%	75.8%	92.2%	80.9%	80.7%
VI	0.0%	0.3%	0.0%	0.0%	0.0%	0.0%	0.0%	0.0%	0.0%	0.0%	0.0%	0.0%

CO	0.0%	51.5%	0.0%	0.0%	100.0%	0.0%	0.0%	7.9%	0.0%	0.0%	0.0%	0.0%
dCO	0.0%	48.5%	0.0%	0.0%	0.0%	0.0%	0.0%	92.1%	0.0%	0.0%	0.0%	0.0%

C16:0	6.9%	0.0%	0.0%	0.0%	0.0%	6.9%	0.0%	0.0%	10.4%	0.8%	11.6%	0.8%
C16:1	31.0%	0.0%	0.0%	19.8%	0.0%	20.5%	12.7%	0.0%	41.8%	10.5%	23.6%	29.6%
C16:2	0.0%	0.0%	0.0%	13.3%	0.0%	1.1%	0.0%	0.0%	0.0%	0.0%	0.0%	0.0%
C18:0	8.0%	0.0%	0.0%	29.0%	0.0%	16.1%	35.1%	0.0%	5.0%	27.0%	0.9%	12.4%
C18:1	28.7%	0.0%	0.0%	0.0%	0.0%	30.3%	28.3%	0.0%	42.8%	36.0%	42.7%	32.4%
C18:2	0.0%	0.0%	0.0%	0.0%	0.0%	2.9%	0.0%	0.0%	0.0%	0.0%	7.1%	0.0%
C20:0	0.0%	0.0%	0.0%	0.0%	0.0%	1.1%	0.0%	0.0%	0.0%	0.0%	3.0%	0.0%
C20:1	0.0%	0.0%	0.0%	18.6%	0.0%	8.5%	0.0%	0.0%	0.0%	22.9%	3.2%	13.9%
C20:2	25.3%	0.0%	0.0%	19.3%	0.0%	12.7%	23.9%	0.0%	0.0%	2.7%	7.9%	10.9%

no methyl	0.0%	54.5%	0.0%	0.0%	100.0%	100.0%	0.0%	18.4%	0.0%	0.0%	0.0%	0.0%
methyl	100.0%	45.5%	0.0%	100.0%	0.0%	0.0%	100.0%	81.6%	100.0%	100.0%	100.0%	100.0%

no fucose	9.1%	49.8%	0.0%	94.6%	54.4%	17.8%	0.0%	15.6%	17.0%	0.0%	6.7%	9.7%
fucose	90.9%	50.2%	0.0%	5.4%	45.6%	82.2%	100.0%	84.4%	83.0%	100.0%	93.3%	90.3%

no carbamoyl	19.9%	60.2%	0.0%	10.8%	100.0%	68.7%	100.0%	24.7%	6.7%	12.9%	20.4%	12.6%
carbamoyl	80.1%	39.8%	0.0%	89.2%	0.0%	31.3%	0.0%	75.3%	93.3%	87.1%	79.6%	87.4%

no arabinose	48.3%	100.0%	0.0%	99.1%	100.0%	58.7%	57.1%	83.2%	75.3%	100.0%	72.2%	83.8%
arabinose	51.7%	0.0%	0.0%	0.9%	0.0%	41.3%	42.9%	16.8%	24.7%	0.0%	27.8%	16.2%
