# Supplementary material for: New insights into Nod factor biosynthesis: Analyses of chitooligomers and lipo-chitooligomers of Rhizobium sp. IRBG74 mutants
Source: Carbohydr Res. 2016 Nov 3;434:83–93. doi: 10.1016/j.carres.2016.08.001 (PMC5080398; doi:10.1016/j.carres.2016.08.001)
Supplement: Supplementary file 1 [file mmc1.pdf]

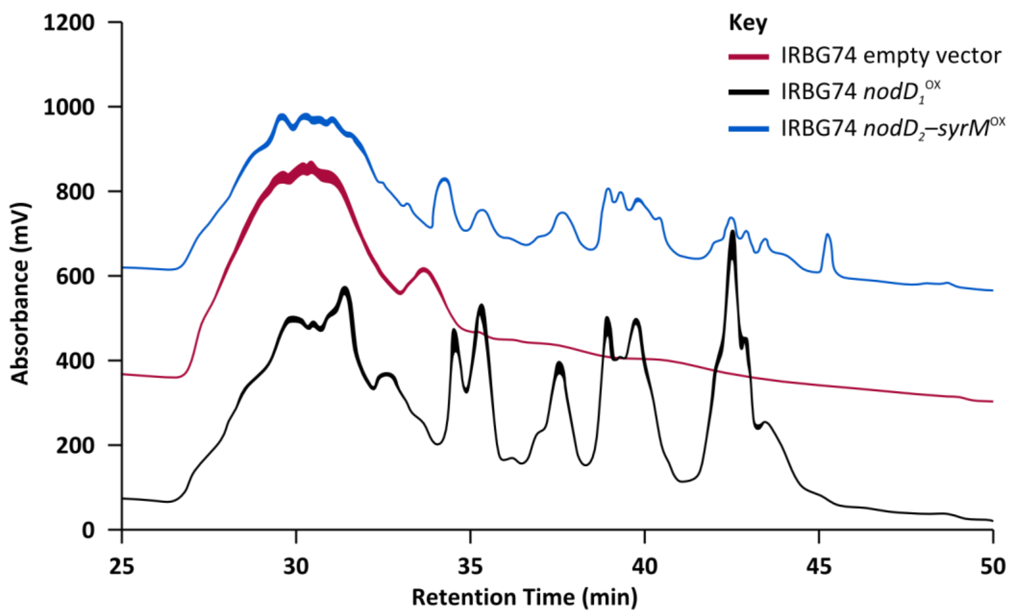

Figure S1. HPLC chromotographs of *Rhizobium* sp. IRBG74 transformed with an empty vector, a *nodD*$_1$ overexpression vector, or a *nodD*$_2$*–syrM* overexpression vector and induced with apigenin.

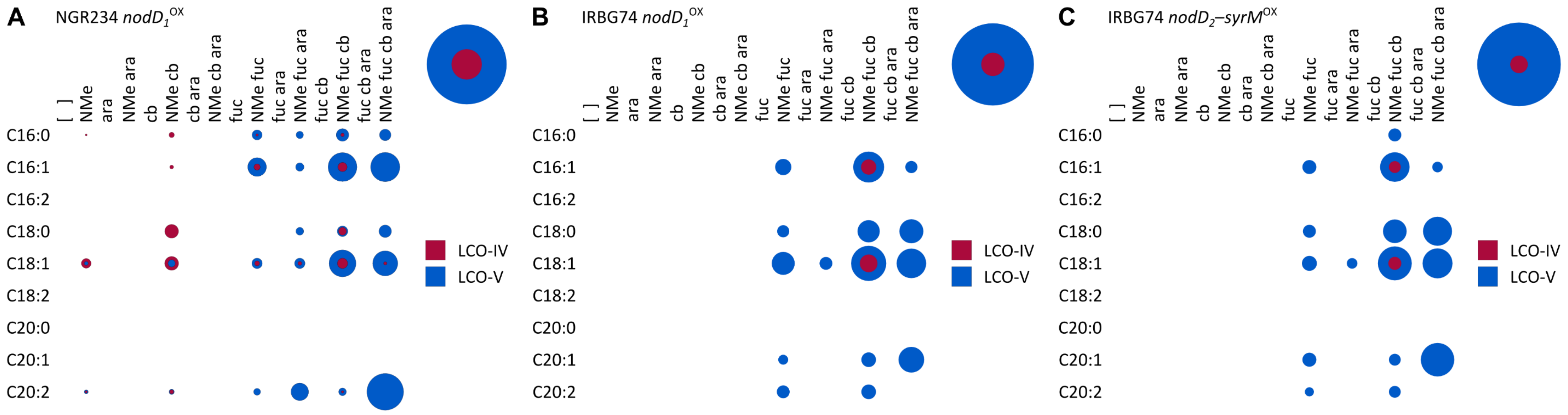

Supplemental data, Figure S2. Bubble matrices of LCOs produced by *Rhizobium* sp. IRBG74 overexpressing **A.** the *nodD*$_1$ gene of *Sinorhizobium* sp. NGR234, **B.** its own *nodD*$_1$ gene, or **C.** its own *nodD*$_2$–*syrM* expression module. ara, arabinose; cb, carbamoyl; fuc, fucose; NMe, *N*-methyl. Relative abundances of different chemical species are represented by the size of the bubble where a given row and column overlap. The bubbles at the top right of the figure indicate the overall abundances of the indicated classes of chemicals using the same scale. Figure S3 follows this same format.

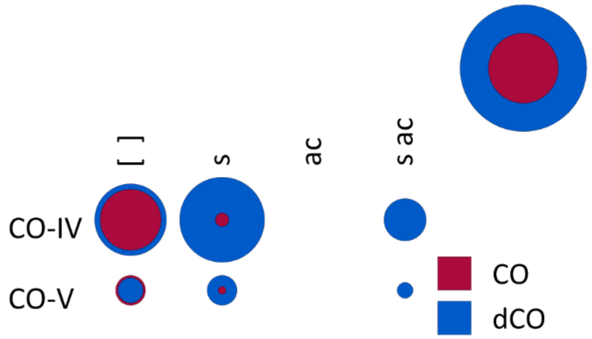

Supplemental data, Figure S3. A bubble matrix of COs and dCOs produced by a *nodA* mutant of *Sinorhizobium meliloti* 1021. ac, acetyl; s, sulfate.

Supplemental data, Table S1. Strains and plasmids used in this study.

Strain	Description[a]	Source
B001	*Escherichia coli* DH5α harboring helper plasmid pRK600 for triparental mating; Cm^R	[1]
IRBG74GUS	*Rhizobium* sp. IRBG74GUS; Sp^R	[2]
MC302	*Rhizobium* sp. IRBG74GUS Δ*nodA*; Sp^R	this study
MC333	*Rhizobium* sp. IRBG74GUS Δ*nodB*; Sp^R	this study
MC512	*Rhizobium* sp. IRBG74GUS Δ*nodC*; Sp^R	this study
MC655	*Rhizobium* sp. IRBG74GUS Δ*nodABC*; Sp^R	this study
MC606	*Rhizobium* sp. IRBG74GUS Δ*nodE*; Sp^R	this study
MC526	*Rhizobium* sp. IRBG74GUS Δ*nodS*; Sp^R	this study
MC334	*Rhizobium* sp. IRBG74GUS Δ*nodU*; Sp^R	this study
MC314	*Rhizobium* sp. IRBG74GUS Δ*nodZ*; Sp^R	this study
MC301	*Rhizobium* sp. IRBG74GUS Δ*noeP*; Sp^R	this study
MC656	*Rhizobium* sp. IRBG74GUS Δ*noeN*; Sp^R	this study
MC642	*Rhizobium* sp. IRBG74GUS Δ*IRBLv2_p0345*; Sp^R	this study
MC152	*Rhizobium* sp. IRBG74GUS harboring pA28; Sp^R, Tc^R	this study
MC315	*Rhizobium* sp. IRBG74GUS Δ*nodA* harboring pA28; Sp^R, Tc^R	this study
MC347	*Rhizobium* sp. IRBG74GUS Δ*nodB* harboring pA28; Sp^R, Tc^R	this study
MC536	*Rhizobium* sp. IRBG74GUS Δ*nodC* harboring pA28; Sp^R, Tc^R	this study
MC703	*Rhizobium* sp. IRBG74GUS Δ*nodABC* harboring pA28; Sp^R, Tc^R	this study
MC638	*Rhizobium* sp. IRBG74GUS Δ*nodE* harboring pA28; Sp^R, Tc^R	this study
MC540	*Rhizobium* sp. IRBG74GUS Δ*nodS* harboring pA28; Sp^R, Tc^R	this study
MC348	*Rhizobium* sp. IRBG74GUS Δ*nodU* harboring pA28; Sp^R, Tc^R	this study
MC316	*Rhizobium* sp. IRBG74GUS Δ*nodZ* harboring pA28; Sp^R, Tc^R	this study

MC317	*Rhizobium* sp. IRBG74GUS Δ*noeP* harboring pA28; Sp^R, Tc^R	this study
MC704	*Rhizobium* sp. IRBG74GUS Δ*noeN* harboring pA28; Sp^R, Tc^R	this study
MC628	*Rhizobium* sp. IRBG74GUS Δ*IRBLv2_p0345* harboring pA28; Sp^R, Tc^R	this study
MC305	*Rhizobium* sp. IRBG74GUS harboring pMBC242; Sp^R, Tc^R	this study
MC306	*Rhizobium* sp. IRBG74GUS harboring pMBC243; Sp^R, Tc^R	this study
GMI3253	*Sinorhizobium meliloti* 1021 Δ*nodA*; Sm^R	[3]
GMI5389	*Sinorhizobium meliloti* 2011 *nodC*::Tn*5*; Sm^R	[4]
GMI3264	*Sinorhizobium meliloti* 1021 Δ*nodA* harboring pMH682; Sm^R	this study
GMI6690	*Sinorhizobium meliloti* 2011 *nodC*::Tn*5* harboring pMH682; Sm^R	this study

**Plasmid**	**Description**	**Source**
pRK600	Self-transmissible helper plasmid; Cm^R	[5]
pA28	pRK7813 expressing *nodD*$_1$ of *Sinorhizobium* sp. NGR234 from P_{trp}; Tc^R	[6]
pMH682	pWB5A expressing *syrM–nodD*$_3$ of *Sinorhizobium meliloti* 2011; Tc^R	[7]
pJQ200SK	Suicide vector for construction of *sacB*-mediated deletions; Gm^R	[8]
pMBC219	pJQ200SK with the Gateway® cassette cloned into XbaI; Cm^R, Gm^R	this study
pMBC229	deletion fragments for *nodA* cloned into pJQ200SK; Gm^R	this study
pMBC248	deletion fragments for *nodB* cloned into pJQ200SK; Gm^R	this study
pMBC279	deletion fragments for *nodC* cloned into pJQ200SK; Gm^R	this study
pMBC419	deletion fragments for *nodABC* cloned into pJQ200SK; Gm^R	this study
pMBC281	deletion fragments for *nodE* cloned into pJQ200SK; Gm^R	this study
pMBC280	deletion fragments for *nodS* cloned into pJQ200SK; Gm^R	this study
pMBC249	deletion fragments for *nodU*cloned into pJQ200SK; Gm^R	this study

pMBC231	deletion fragments for *nodZ* cloned into pJQ200SK; Gm^R	this study
pMBC230	deletion fragments for *noeP* cloned into pJQ200SK; Gm^R	this study
pMBC420	deletion fragments for *noeN* cloned into pJQ200SK; Gm^R	this study
pMBC401	deletion fragments for *IRBLv2_p0345* cloned into pJQ200SK; Gm^R	this study
pRF771	RK2-derived P_{trp} expression plasmid similar to pRK7813; Tc^R	[9]
pMBC223	pRF771 with the Gateway® cassette cloned into XbaI; Cm^R, Tc^R	this study
pMBC242	*nodD₁* of *Rhizobium* sp. IRBG74 cloned into pRF771; Tc^R	this study
pMBC243	*nodD₂–syrM* of *Rhizobium* sp. IRBG74 cloned into pRF771; Tc^R	this study

[a] Cm^R, chloramphenicol resistance; Gm^R, gentamicin resistance; Sm^R, streptomycin resistance; Sp^R, spectinomycin resistance; Tc^R, tetracycline resistance

Supplemental data, Table S2. Primers used in this study.

Name	Sequence[a,b]	Direction	Purpose
oMBC032	CGCTCTAGAACAAGTTTGTACAAAAAAGCTG	forward	clone the pDEST24 Gateway® cassette into pJQ200SK and pRF771
oMBC049	CGCTCTAGACCACTTTGTACAAGAAAGC	reverse	clone the pDEST24 Gateway® cassette into pJQ200SK and pRF771
oMBC001	CGACTCACTATAGGGCGAATTG	forward	sequence verification of pJQ200SK inserts
oMBC002	ACAGGAAACAGCTATGACCATG	reverse	sequence verification of pJQ200SK inserts
oMBC056	CACCGATCCCAATTGCGATCTCG	forward	in-frame deletion of *nodA* of *Rhizobium* sp. IRBG74
oMBC004	tcatagctctggCACGTCAGAACACATTTCATCT	reverse	in-frame deletion of *nodA* of *Rhizobium* sp. IRBG74
oMBC005	tgttctgacgtgCCAGAGCTATGAAACAGCTG	forward	in-frame deletion of *nodA* of *Rhizobium* sp. IRBG74
oMBC057	TGGCTGAACTGAATTCAGC	reverse	in-frame deletion of *nodA* of *Rhizobium* sp. IRBG74
oMBC007	CTCAATGCGCATGGCACTGG	forward	check primer for *Rhizobium* sp. IRBG74 *nodA* deletion
oMBC008	GAACCTGCATGCCTTTATAGAC	reverse	check primer for *Rhizobium* sp. IRBG74 *nodA* deletion
oMBC107	CACCGTTCTTTCGGAATACCTATGG	forward	in-frame deletion of *nodB* of *Rhizobium* sp. IRBG74
oMBC108	cctttaattgtgCAGCTGTTTCATAGCTCTGG	reverse	in-frame deletion of *nodB* of *Rhizobium* sp. IRBG74
oMBC109	atgaaacagctgCACAATTAAAGGACGGTATTGC	forward	in-frame deletion of *nodB* of *Rhizobium* sp. IRBG74
oMBC110	CTATCGATGGATCGCGCATC	reverse	in-frame deletion of *nodB* of *Rhizobium* sp. IRBG74
oMBC111	CAAGGGATGAATGCGTTTCATC	forward	check primer for *Rhizobium* sp. IRBG74 *nodB* deletion
oMBC112	GTCTCGTATTGGTCGAGCAG	reverse	check primer for *Rhizobium* sp. IRBG74 *nodB* deletion
oMBC173	CACCAGCACTTATCCAACGTATGG	forward	in-frame deletion of *nodC* of *Rhizobium* sp. IRBG74
oMBC174	ttaatccatcggACCAAATAGATCCATGCAATACC	reverse	in-frame deletion of *nodC* of *Rhizobium* sp. IRBG74
oMBC175	gatctatttggtCCGATGGATTAACCTTTTCGCG	forward	in-frame deletion of *nodC* of *Rhizobium* sp. IRBG74
oMBC176	CATTTCAGCAACGCTCTCAAGG	reverse	in-frame deletion of *nodC* of *Rhizobium* sp. IRBG74
oMBC177	CTGATTGATCGTAATGGCCCAG	forward	check primer for *Rhizobium* sp. IRBG74 *nodC* deletion
oMBC178	GTAAGCAAACAGTCTTCGTTGG	reverse	check primer for *Rhizobium* sp. IRBG74 *nodC* deletion

oMBC056	CACCGATCCCAATTGCGATCTCG	forward	in-frame deletion of *nodABC* of *Rhizobium* sp. IRBG74
oMBC213	ttaatccatcggCACGTCAGAACACATTTCATCT	reverse	in-frame deletion of *nodABC* of *Rhizobium* sp. IRBG74
oMBC214	tgttctgacgtgCCGATGGATTAACCTTTTCGCG	forward	in-frame deletion of *nodABC* of *Rhizobium* sp. IRBG74
oMBC176	CATTTCAGCAACGCTCTCAAGG	reverse	in-frame deletion of *nodABC* of *Rhizobium* sp. IRBG74
oMBC007	CTCAATGCGCATGGCACTGG	forward	check primer for *Rhizobium* sp. IRBG74 *nodABC* deletion
oMBC178	GTAAGCAAACAGTCTTCGTTGG	reverse	check primer for *Rhizobium* sp. IRBG74 *nodABC* deletion
oMBC185	CACCGCATAGCAACAGCAACTGACG	forward	in-frame deletion of *nodE* of *Rhizobium* sp. IRBG74
oMBC186	ctatacttgcctCCCATCCATTCAAGCCTCCT	reverse	in-frame deletion of *nodE* of *Rhizobium* sp. IRBG74
oMBC187	tgaatggatgggAGGCAAGTATAGCAACGGCAG	forward	in-frame deletion of *nodE* of *Rhizobium* sp. IRBG74
oMBC188	CCTCGAAGGGCTGTGCATTG	reverse	in-frame deletion of *nodE* of *Rhizobium* sp. IRBG74
oMBC189	CAAACGCGATCTGCGCTTCC	forward	check primer for *Rhizobium* sp. IRBG74 *nodE* deletion
oMBC190	CATTGAATGACTAAGCCCAAGC	reverse	check primer for *Rhizobium* sp. IRBG74 *nodE* deletion
oMBC179	CACCATTGCAGCTACAGTCGTGC	forward	in-frame deletion of *nodS* of *Rhizobium* sp. IRBG74
oMBC180	tcattgagaaacCAGCTGCAACATCCAAAGCC	reverse	in-frame deletion of *nodS* of *Rhizobium* sp. IRBG74
oMBC181	atgttgcagctgGTTTCTCAATGATCCGGCTACC	forward	in-frame deletion of *nodS* of *Rhizobium* sp. IRBG74
oMBC182	AGCCATCCCATACCAAGCAG	reverse	in-frame deletion of *nodS* of *Rhizobium* sp. IRBG74
oMBC183	GTGATGTGCTGTTGTGGTCC	forward	check primer for *Rhizobium* sp. IRBG74 *nodS* deletion
oMBC184	GCTCCCGAAACACGTTTAGG	reverse	check primer for *Rhizobium* sp. IRBG74 *nodS* deletion
oMBC113	CACCGCAGCAATCCATCACACACG	forward	in-frame deletion of *nodU* of *Rhizobium* sp. IRBG74
oMBC016	gcgtgtcgtatgCGTGAGTTTAATTCCGCAAATG	reverse	in-frame deletion of *nodU* of *Rhizobium* sp. IRBG74
oMBC017	attaaactcacgCATACGACACGCCCGGAATG	forward	in-frame deletion of *nodU* of *Rhizobium* sp. IRBG74
oMBC114	GAAGCACTCTCCTTTTTTGACG	reverse	in-frame deletion of *nodU* of *Rhizobium* sp. IRBG74
oMBC115	GCTTTGGATGTTGCAGCTGAC	forward	check primer for *Rhizobium* sp. IRBG74 *nodU* deletion
oMBC020	GTTCTCGCGCACTGTGAATTC	reverse	check primer for *Rhizobium* sp. IRBG74 *nodU* deletion
oMBC075	CACCGAATGCTGGCTGAAGCTGC	forward	in-frame deletion of *nodZ* of *Rhizobium* sp. IRBG74

oMBC010	ctttgacactccGTCAGCAAGCATTTGCGGTG	reverse	in-frame deletion of *nodZ* of *Rhizobium* sp. IRBG74
oMBC011	atgcttgctgacGGAGTGTCAAAGGATCTCTG	forward	in-frame deletion of *nodZ* of *Rhizobium* sp. IRBG74
oMBC076	CTCACCTGGACATGGCTTTG	reverse	in-frame deletion of *nodZ* of *Rhizobium* sp. IRBG74
oMBC013	TCGTCCGCACCGAAAACTGC	forward	check primer for *Rhizobium* sp. IRBG74 *nodZ* deletion
oMBC014	CATTTTGCGGGACTTCTTGAGC	reverse	check primer for *Rhizobium* sp. IRBG74 *nodZ* deletion
oMBC060	<u>CACC</u>ATGCGTAGCAACTACGAAGG	forward	in-frame deletion of *noeP* of *Rhizobium* sp. IRBG74
oMBC061	ctccaaataaccCCATATCAGGCCGAACATTCC	reverse	in-frame deletion of *noeP* of *Rhizobium* sp. IRBG74
oMBC062	ggcctgatatggGGTTATTTGGAGTGAGTTGTGG	forward	in-frame deletion of *noeP* of *Rhizobium* sp. IRBG74
oMBC063	GAGGATGTCGTTCAGACATTGG	reverse	in-frame deletion of *noeP* of *Rhizobium* sp. IRBG74
oMBC064	CAATCAGAAACACTCGATGCTG	forward	check primer for *Rhizobium* sp. IRBG74 *noeP* deletion
oMBC065	CAGCTCCGCAACAAGGTTCC	reverse	check primer for *Rhizobium* sp. IRBG74 *noeP* deletion
oMBC026	GACATTTGCATTACACGGTTGG	forward	sequence verification of pRF771 inserts
oMBC232	<u>CACC</u>GACCACCGCCTATTCCCTG	forward	in-frame deletion of *noeN* of *Rhizobium* sp. IRBG74
oMBC233	agaaatccttctGTTTCACCTCAGCACTCCG	reverse	in-frame deletion of *noeN* of *Rhizobium* sp. IRBG74
oMBC234	ctgaggtgaaacAGAAGGATTTCTTCATGAAGCTCT	forward	in-frame deletion of *noeN* of *Rhizobium* sp. IRBG74
oMBC235	GGATAATGATCCCAGTCAGC	reverse	in-frame deletion of *noeN* of *Rhizobium* sp. IRBG74
oMBC236	CAGCTTGGTGGCTTCTAGC	forward	check primer for *Rhizobium* sp. IRBG74 *noeN* deletion
oMBC237	GAGGTGTTCGCGTGATCTCC	reverse	check primer for *Rhizobium* sp. IRBG74 *noeN* deletion
oMBC191	<u>CACC</u>AACCAACAACGCGGTACC	forward	in-frame deletion of *IRBLv2_p0345* of *Rhizobium* sp. IRBG74
oMBC192	gttggtcggtgtAGTTCGTGCCATATCGAAACTTG	reverse	in-frame deletion of *IRBLv2_p0345* of *Rhizobium* sp. IRBG74
oMBC193	atggcacgaactACACCGACCAACTGAGGAGG	forward	in-frame deletion of *IRBLv2_p0345* of *Rhizobium* sp. IRBG74
oMBC194	GAGCCGAACTCAGTTACTGC	reverse	in-frame deletion of *IRBLv2_p0345* of *Rhizobium* sp. IRBG74
oMBC195	CCTTCTTTCGGATTTCATGACG	forward	check primer for *Rhizobium* sp. IRBG74 *IRBLv2_p0345* deletion
oMBC196	CGTTCTCTTGAATACCGTGGAG	reverse	check primer for *Rhizobium* sp. IRBG74 *IRBLv2_p0345* deletion
oMBC027	ACCTGCGTTCAGCAGTTCTG	reverse	sequence verification of pRF771 inserts

oMBC096	CACCTCTTCATGAGCTTCGAAGTC	forward	clone *nodD*$_1$ of *Rhizobium* sp. IRBG74
oMBC097	CGACGGAAAGTACAGATCTCG	reverse	clone *nodD*$_1$ of *Rhizobium* sp. IRBG74
oMBC098	CACCTATCTGATCCCAGTGTTATCC	forward	clone *nodD*$_2$–*syrM* of *Rhizobium* sp. IRBG74
oMBC099	CTTGGCGGTTTGTCCTGTTAC	reverse	clone *nodD*$_2$–*syrM* of *Rhizobium* sp. IRBG74

[a]Underlined sequences indicate restriction sites or TOPO® recognition sites (for Gateway® cloning)

[b]Lowercase letters indicate primer overlaps used during overlap-extension PCR

Supplemental References

1. Griffitts, J.S.; Carlyon, R.E.; Erickson, J.H.; Moulton, J.L.; Barnett, M.J.; Toman, C.J.; Long, S.R. *Mol. Microbiol.*, **2008**, *69(2)*, 479–90. http://dx.doi.org/10.1111/j.1365-2958.2008.06304.x.
2. Cummings, S.P.; Gyaneshwar, P.; Vinuesa, P.; Farruggia, F.T.; Andrews, M.; Humphry, D.; Elliott, G.N.; Nelson, A.; Orr, C.; Pettitt, D.; Shah, G.R.; Santos, S.R.; Krishnan, H.B.; Odee, D.; Moreira, F.M.S.; Sprent, J.I.; Young, J.P.W.; James, E.K. *Environ. Microbiol.*, **2009**, *11(10)*, 2510–25. http://dx.doi.org/10.1111/j.1462-2920.2009.01975.x.
3. Debellé, F.; Plazanet, C.; Roche, P.; Pujol, C.; Savagnac, a.; Rosenberg, C.; Promé, J.C.; Dénarié, J. *Mol. Microbiol.*, **1996**, *22(2)*, 303–14. http://dx.doi.org/10.1046/j.1365-2958.1996.00069.x.
4. Debellé, F.; Rosenberg, C.; Vasse, J.; Maillet, F.; Martínez, E.; Dénarié, J.; Truchet, G. *J. Bacteriol.*, **1986**, *168(3)*, 1075–86.
5. Finan, T.M.; Kunkel, B.; De Vos, G.F.; Signer, E.R. *J. Bacteriol.*, **1986**, *167(1)*, 66–72. http://dx.doi.org/0021-9193/86/070066-05$02.00/0.
6. Price, N.P.J.; Relić, B.; Talmont, F.; Lewin, A.; Promé, D.; Pueppke, S.G.; Maillet, F.; Dénarié, J.; Promé, J.-C.; Broughton, W.J. *Mol. Microbiol.*, **1992**, *6(23)*, 3575–84. http://dx.doi.org/doi:10.1111/j.1365-2958.1992.tb01793.x.
7. Honma, M.A.; Asomaning, M.; Ausubel, F.M. *J. Bacteriol.*, **1990**, *172(2)*, 901–11.
8. Quandt, J.; Hynes, M.F. *Gene*, **1993**, *127(1)*, 15–21. http://dx.doi.org/10.1016/0378-1119(93)90611-6.
9. Wells, D.H.; Long, S.R. *Mol. Microbiol.*, **2002**, *43(5)*, 1115–27. http://dx.doi.org/10.1046/j.1365-2958.2002.02826.x.
